# HPV and HIV Coinfection in Women from a Southeast Region of Romania—PICOPIV Study

**DOI:** 10.3390/medicina58060760

**Published:** 2022-06-03

**Authors:** Simona Claudia Cambrea, Mariana Aschie, Ghiulendan Resul, Anca Florentina Mitroi, Anca Chisoi, Antonela Anca Nicolau, Gabriela Izabela Baltatescu, Ana Maria Cretu, Gabriela Lupasteanu, Lucian Serbanescu, Mihaela Manea, Sebastian Theodor Topliceanu, Lucian Cristian Petcu, Loredana Pazara, Georgeta Camelia Cozaru

**Affiliations:** 1 Faculty of Medicine, Ovidius University of Constanta, 1 Universitatii Street, 900470 Constanta, Romania; cambrea.claudia@gmail.com (S.C.C.); aschiemariana@yahoo.com (M.A.); lucian_trocadero@yahoo.com (L.S.); loredanapazara@yahoo.com (L.P.); 2 Clinical Hospital of Infectious Diseases, 100 Ferdinand Blvd., 900178 Constanta, Romania; 3 Center for Research and Development of the Morphological and Genetic Studies of Malignant Pathology, Ovidius University of Constanta, 145 Tomis Blvd., 900591 Constanta, Romania; ank_mitroi@yahoo.com (A.F.M.); aka_dobre@yahoo.com (A.C.); ancanicolau@rocketmail.com (A.A.N.); gabrielabaltatescu@yahoo.com (G.I.B.); cretu_anamaria@yahoo.com (A.M.C.); mihaela.manea@sunmedical.ro (M.M.); topliceanu.sebastian@gmail.com (S.T.T.); drcozaru@yahoo.com (G.C.C.); 4 Institute of Doctoral Studies, Ovidius University of Constanta, 1 Universitatii Street, 900470 Constanta, Romania; 5 Sf. Apostol Andrei Emergency County Hospital, 145 Tomis Blvd., 900591 Constanta, Romania; 6 Clinical Hospital of Infectious Disease Sf. Cuvioasa Parascheva, 393 Traian Street, 800179 Galati, Romania; gabidoana@yahoo.com; 7 Faculty of Dentistry, Ovidius University of Constanta, 1 Universitatii Street, 900470 Constanta, Romania; crilucpetcu@gmail.com

**Keywords:** HIV, HPV, high risk, coinfection, cervical precancerous lesions, pap cytology, CD4, antiretroviral therapy

## Abstract

*Background and Objectives*: Romania faces one of the highest cervical cancer burdens in Europe though it is a preventable cancer through population screening by cytology and human papillomavirus (HPV) detection. Also, it has one of the highest incidences of human immunodeficiency virus (HIV) infection. HPV and HIV coinfection are frequently encountered. The aim of study was to establish the prevalence of HPV infection among HIV-positive women in Southeast Region of Romania, to genotype high risk HPV types -and to correlate the results with clinical data and cytological cervical lesions. *Materials and Methods*: 40 HIV-positive women were screened for HPV types and for cytological cervical lesions. The findings were evaluated in correlation with CD4 cell counts, HIV viral load, age at first sexual intercourse, number of sexual partners, vaginal candidiasis, and Gardnerella using statistical methods. *Results*: 19/40 (47.5%) women were positive for HPV types, 63.15% infected with single HPV type and 36.85% with multiple HPV types. The most frequent types were type: 31 (42.1%), 56 (31.57%), 53 (15.78%). On cytology, 34 (85%) women were found with NILM of which 38.23% were HPV-positive. Fifteen percent of women had abnormal cytology (three ASC-US, three LSIL), and all of them were HPV-positive. Through analyzing the value of CD4 count, women with CD4 count ≤ 200 cells/μL were found to be significantly more likely to be infected with HPV; meanwhile there was no correlation between the detection of HPV types and HIV viral load. Candida or Gardnerella were more often associated with HIV-positive women with HPV, than in women without HPV. *Conclusions:* Infection with HPV types is common among HIV-positive women in the Southeast Region of Romania and it is associated with age at the beginning of sexual life, number of sexual partners, CD4 value, vaginal candidiasis, and Gardnerella infection.

## 1. Introduction

HPV infection is the most common sexually transmitted infection in young women. Its natural evolution is to regression and to spontaneous clearance in case of a competent immune system. According to the study of Ho et al. (1998), HPV infection may regress spontaneously within one year in 70% of cases and in 24 months in 91% of them [1].

Over 100 different HPV genotypes have been described, more than 40 of them infecting the anogenital tract. Of these, approximately 1/3 are associated with cervical cancer and anal neoplasm. The anogenital HPV’s are generally divided into two categories regarding their potential risk to develop cancer: low oncogenic types and medium-high oncogenic types. The high-risk HPV (HR-HPV) genotypes are generally associated with high-grade precancerous lesions and invasive cancer; the association between oncogenic HR-HPV types and cervical cancer has been well-established, while low-risk HPVs are frequently found in asymptomatic or benign conditions such as genital warts [2,3,4,5].

Persistent HR-HPV infection is responsible for over 90% of cervical cancers, but prophylactic vaccination against HPV infections including high-risk genotypes can reduce cervical cancers [6].

In HIV infection, premalignant cervical lesions have a higher prevalence, a greater persistence, and a higher tendency to relapse than in the general population [7,8,9].

Cervical cancer is the most common cancer among HIV-positive women, being up to five times higher than in HIV-negative women [10]. Since 1993, cervical cancer is categorized by the Centers for Disease Control and Prevention as an acquired immunodeficiency syndrome-defining illness [11].

This close correlation between HPV and HIV infection is so obvious, leading to absolute international recognition by being awarded with the Nobel Prize in Medicine 2008 at the same time for “the parents” of these two viruses: Harald zur Hausen “for his discovery of human papillomaviruses causing cervical cancer”, and Françoise Barré-Sinoussi and Luc Montagnier “for their discovery of human immunodeficiency virus” [12]. Unlike HIV infection, genital HPV infection is not a reportable disease, so actual incidence and prevalence are not known [2,3]. Romania faces a high prevalence of both HPV and HIV infections.

For years, Romania stands in the first position in the international statistics reports regarding the incidence and mortality of cervical cancer, even from the first year (2012) of implementation of the National Cervical Cancer Screening Program. The increased efforts of cervical cancer screening by cytology determined favorable results, beginning with 2015, when Romania managed to no longer be the leader in international statistics reports [13].

According to the International Agency for Research on Cancer, in 2020, cervical cancer in Romania was the third most prevalent cancer in females, following breast and colorectal cancer in statistics. In Europe, Romania also stands in second place regarding estimated age-standardized incidence rates of cervical cancer for 20–54 aged females [14,15].

The most effective way to prevent cervical cancer is through the Babes-Papanicolau cervicovaginal cytological examination (Pap cytology test) associated with the detection of HR-HPV strains. Although the etiology of cervical cancer is multifactorial, HPV infection is crucial in the vast majority of cases. Regarding the fact that HPV infection is a sexually transmitted disease, it is known that the risk increases with an increasing number of sexual partners and with the early onset of sexual life. For primary prevention of cervical cancer, there is an available HPV vaccine.

It is estimated that three of four HIV-positive women are infected with HPV. The prevalence of HPV infection among women with HIV is directly associated with HIV viral load and CD4 levels [16,17].

Antiretroviral therapy (ART) lowers HIV viral load, CD4 levels increase and it decreases the incidence of opportunistic infections and certain cancers. Studies on the impact of ART on the natural history of premalignant cervical lesions were inconclusive [18,19].

As HPV infection becomes more persistent, its possibility for spontaneous clearance decreases and the probability of evolution towards premalignant lesions or cancer grows [2]. However, cervical cancer is preventable and curable if diagnosed and treated early [20].

In our study, we analyzed the prevalence of HPV infection among HIV-positive women in the Southeast Region of Romania, genotyped HR-HPV types, and correlated them with clinical data and cytological cervical lesions.

## 2. Materials and Methods

### 2.1. Participants, Data Collection and Ethical Statement

Data were obtained by a descriptive study analyzing demographics of HPV and cervical lesions in HIV-positive women from Constanta, Southeast Region of Romania. Between September 2018 and February 2020 (18 months), 40 HIV-positive women undergoing cervical cancer screening were included in the study after considering the inclusion and exclusion criteria.

Samples were collected from the Day Clinic of Constanta’s Clinical Infectious Diseases Hospital. After collection of demographic data, cervical samples were used both for screening cytology of cervical lesions and for determining the HPV strains. Blood samples were collected in order to determine CD4 counts and HIV viral load. Also, medical history was retrospectively retrieved from hospital medical records.

Participation of subjects was entirely voluntary and written informed consent was required for inclusion in the study. All personal data were removed from samples to ensure patient confidentiality. A standardized questionnaire was used to gather demographic data. Other parameters identified and assessed in the questionnaire were time of first sexual intercourse, number of sexual partners, and history of sexually transmitted infections.

### 2.2. Specimen Collection, HIV Serology and Pap Tests

Cervical cytobrush samples were collected from the cervical transformation zone of all women. Samples preservation, transporting, and processing for liquid-based cytology were performed according to the instructions of the reagents manufacturers. For samples preservation transporting Cell Solutions General Preservative (CellSolution LLC, Greensboro, NC, USA, Menarini Diagnostics) was used. Exo-/endocervical cells suspended in fixative liquid were collected by centrifugation (Hettich Zentrifugen Rotofix 32A, Tuttlingen, Germany), transferred in monolayer onto a glass slide and stained with Papanicolaou staining method with Harris’ hematoxylin, Orange G and EA polychromatic solutions according manufacturer’s recommendations (MerckKGaA Darmstadt, Germany). Pap smears were examined by at least three trained cyto/pathologists, independently of HR-HPV results, and classified according to the Bethesda System for Reporting Cervical Cytology (2014): Negative for intraepithelial lesion or malignancy (NILM); atypical squamous cells of undetermined significance (ASC-US); low-grade squamous intraepithelial lesions (LSIL); atypical squamous cells but cannot exclude high-grade lesions (ASC-H); or high-grade squamous intraepithelial lesions (HSIL) [21].

Blood samples were collected by venous puncture in vacutainers, processed according to kit producer and analyzed for CD4 and HIV viral determination. CD4 was determined by lymphocytic immunophenotyping using flow citometry with acoustic focusing cytometer (AB Applied Biosystems Attune, Waltham, MA, USA), reagents from AQUIOS Tetra-1 Panel (Beckman Coulter, Brea, CA, USA). HIV viral load evaluation was performed by Reverse Transcriptase-Polymerase Chain Reaction (RT-PCR) method using 7500 Fast Real TIME Systems (Applied Biosystems, Waltham, MA, USA) and Xpert HIV-1 Viral Load test reagents (Cepheid AB, Solna, Sweden) which include ribonucleic acid (RNA) extraction, purification, reverse transcription, and complementary deoxyribonucleic acid (cDNA) real time quantitation.

### 2.3. HPV Genotyping

Cervical cytobrush samples were vortexed and separated using lysis buffer. DNA was extracted from the lysis buffer using the DNeasy Tissue extraction kit according to the manufacturer’s protocol. (QIAamp^®^ DNA Mini Kit, Qiagen, Redwood City, CA, USA). Using High Papilloma Strip of Operon (Operon, S.A., Cuarte de Huerva, Zaragoza, Spain) qualitative detection of HR-HPV types were made from DNA samples by reverse blot technique. To determine HPV status, DNA samples were genotyped for HR-HPVs (types 16, 18, 30, 31, 33, 35, 39, 45, 51, 52, 53, 56, 58, 59, and 66, 68) using PCR Thermal Cycler (Biometra Product Line Analytic Jena, Jena, Germany).

### 2.4. Statistical Analyses

All the statistical analyses were computed using SPSS Statistics (IBM SPSS Statistics for Windows, Version 23.0, Armonk, NY, USA: IBM Corp.). We computed descriptive statistics (mean, range, and percentage) for continuous variables (age) and inferential statistics with Chi-square for categorical variables. Variance was analyzed using one variable selected as independent (HPV) versus the other remaining dependent variables collected: CD4 level (≤200 cells/μL vs. >200 cells/μL), HIV viral load (<50 copies/mL vs. >51 copies/mL), age at first sexual intercourse (≤18 years vs. >18 years), number of sexual partners (>2 partners vs. ≤2 partners), vaginal candidiasis (yes vs. no), Gardnerella (yes vs. no)). Women aged between 18 and 60 years old, HIV-positive, sexually active, and had signed informed consent were included in the study. We excluded women with a history of cervical precancerous lesions or other malignancies, total hysterectomy, pregnancy, and absence of written informed consent. After inclusion-exclusion criteria, we obtained a group of 40 representative cases. Odds ratios were calculated to identify groups with significantly increased odds of HPV. Significance level was set at *p* < 0.05, two-tailed tested.

## 3. Results

Data of cervical PAP cytology, HPV types, and clinic-epidemiological questionnaire responses are available for 40 HIV-positive women from South-East Romania. The age ranged between 20 and 54 years old and the mean age was 31.77 years old. The statistical analysis was performed on factors presented in Table 1 (CD4, HIV viral load, age at first sexual intercourse, number of sexual partners, vaginal candidiasis, Gardnerella).

### 3.1. HPV Distribution

**Figure 1:**
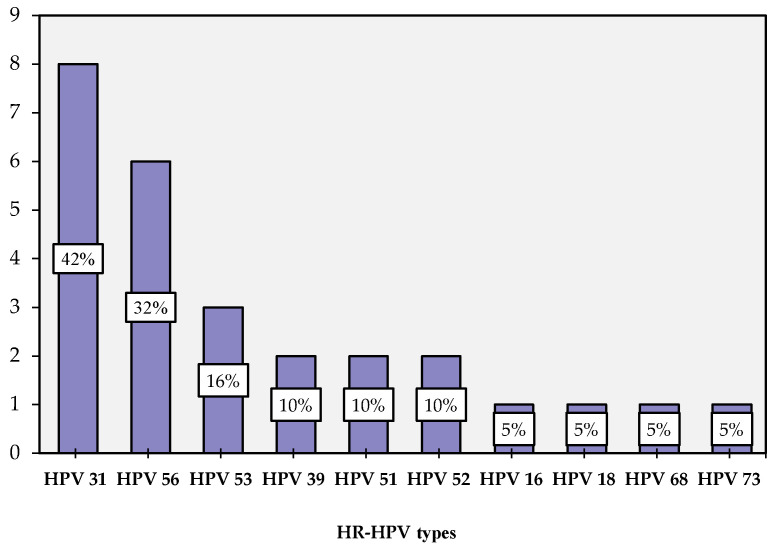
Frequencies of HR-HPV types.

At least one type of HPV was detected in 19 cases (47.5%) including 12 cases with one HPV type (63.15%) and 7 cases (36.85%) with multiple HPV types. The most frequent HPV types were: 31 (8 cases—42.10%), 56 (6 cases—31.57%), 53 (3 cases—15.78%), 39, 51 and 52 HPV types that were identified in 2 (10.52%) cases each. A total of 16, 18, 68 and 73 HPV types were detected in 1 (5.26%) case each (Figure 1).

### 3.2. PAP Cytology

From the total of 40 studied cases, 34 women had no cytological abnormalities and were reported as NILM, while 6 presented cytological lesions.

**Figure 2:**
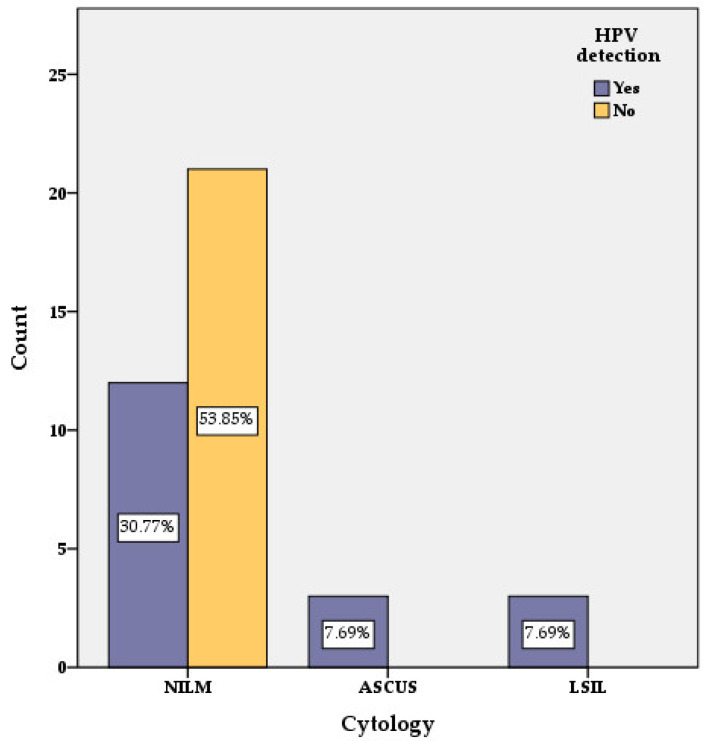
HPV distribution regarding cytology findings.

**Figure 3:**
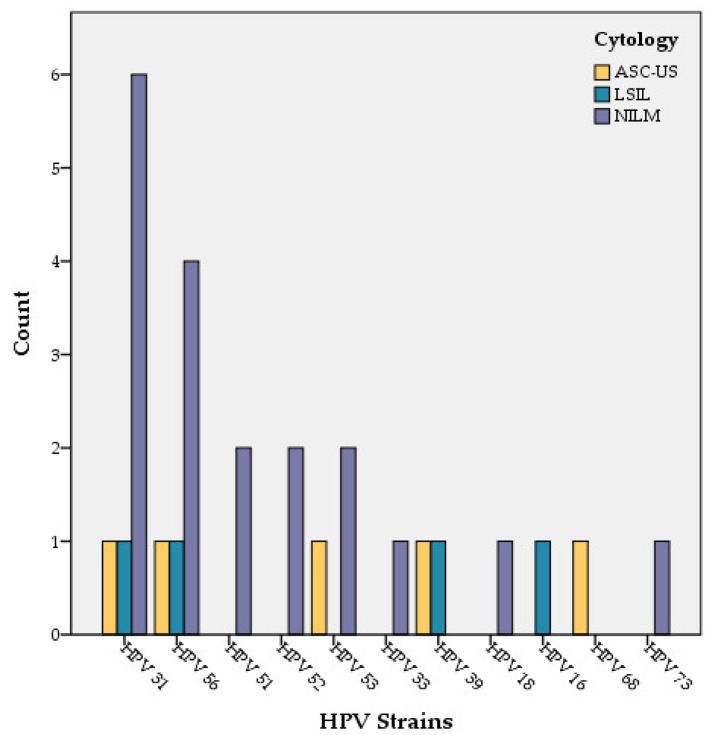
Cytology findings in different HR-HPV types.

In the HPV-positive group (19 out of 40 cases) 13 women were reported as NILM. In this group of HPV-positive women, six had cytological lesions (three of them with ASC-US and three with LSIL) (Figure 2 and Figure 3). In the group of women reported as NILM, four women had multiple HPV types (Table 2).

In the group of HPV-negative women (21 cases out of 40) no cytological abnormalities were identified and they were reported as NILM.

Of 33 cases with CD4 > 200 cells/μL, 4 cases were reported with abnormal Pap cytology (2 with ASC-us and 2 with LSIL) and the rest of them (29 cases) were reported as NILM.

In the CD4 ≤ 200 cells/μL group two cases with abnormal Pap cytology were reported: one with ASC-US and one with LSIL.

### 3.3. Correlation of HPV with CD4 Counts and HIV Viral Load

**Figure 4:**
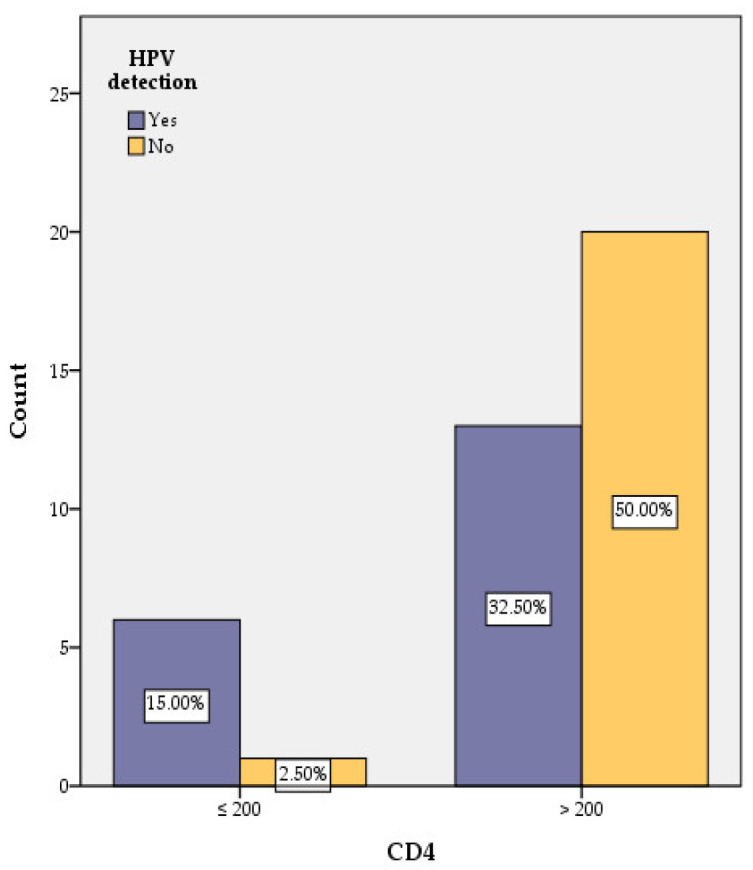
Correlation of HPV with CD4 counts.

Forty HIV-positive women were tested for HPV presence (19 were found positive and 21 negative) and level of CD4 (7 were found with CD4 ≤ 200 cells/μL and 33 with CD4 > 200 cells/μL) (Table 1, Figure 4). A link between the level of CD4 and the presence of HPV was identified.

In the group of HPV-negative cases (21 women, representing 52.5% of all studied cases), only one case had CD4 ≤ 200 cells/μL.

In the group of HPV-positive patients (19 women representing 47.5% of all studied cases) only 6 (31.57% of women out of 19 positive cases) had CD4 ≤ 200 cells/μL.

In the group of CD4 > 200 cells/μL (33 out of 40 cases), 20 women were HPV negative. In the same group (CD4 > 200 cells/μL) 11 cases had only one type of HPV, while 2 cases had 2 types of HPV (Table 1).

Women with CD4 count ≤ 200 cells/μL are significantly more likely to be infected with HPV, with a nine times higher risk compared with the group with CD4 > 200 cells/μL (*p* < 0.05) (Figure 4, Table 1).

**Figure 5:**
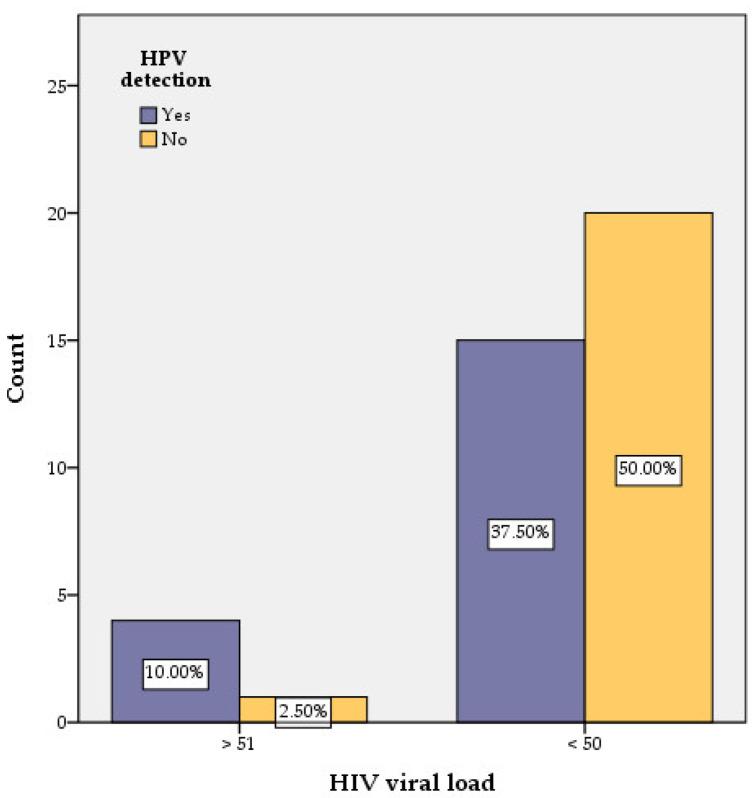
Correlation of HPV with HIV viral load.

The value of HIV viral load in HIV-positive women was another parameter checked for any correlation with HR-HPV presence. Only one out of 21HPV-negativewomen had a detectable HIV viral load, while in the group of HPV-positive women, 4 out of 19 had a detectable HIV viral load (Figure 5, Table 1). Our findings have no statistical significance within our sample size (*p* > 0.05).

### 3.4. Correlation of HPV with Antiretroviral Therapy and Duration of HIV Detection

Analyzing the duration of antiretroviral therapy for HPV-positive and HPV-negative women, we found that the mean duration of therapy for HPV-positive was 12 years, while the mean duration of antiretroviral therapy for the HPV-negative group was 19 years with a statistically significant difference between the two groups (*p* < 0.001). The duration of HIV infection for the group of HPV-positive women was between 13 and 27 years, with a median value of 20 years and an interquartile range = 8 years, and the duration of HIV infection for the HPV-negative group was between 6 and 29 years, with a median value of 19 years and an interquartile range = 10.50 years. By comparing the mean values of the duration of HIV infection between the two groups of HPV-positive and HPV-negative women, we found that there are no significant differences between their values (*p* > 0.05).

### 3.5. Correlation of HPV with Sexual Activity (Number of Sexual Partners and Age at First Sexual Intercourse)

Number of sexual partners was an important factor for the detection of HR-HPV types. Women with two or more previous sex partners were predisposed to HPV infection. The risk of having HPV was greater than 4.13 times in the group of women with more than two sexual partners, compared to the group of women with one or two sexual partners. These differences have statistical significance (*p* < 0.05).

The younger the age when sexual life begins, the higher the risk of being detected with HPV types. Fourteen of the 19 women with detectable HPV types began their sexual life before the age of 18. Only 8 of the 21 patients with undetectable HPV types started having sex before the age of 18. The risk that a woman who began her sexual life before the age of 18 would develop an HPV infection was 4.55 times higher compared to women who began asexual life after 18 years old. The differences between the two groups of patients have statistical significance (*p* < 0.05).

### 3.6. Correlation of HPV with Cytobacteriological Results

Vaginal discharge did not influence the detection of HPV in HIV-positive women but the identification of Candida in vaginal discharge presented a 5.4 times higher risk of having an HPV infection than the women without Candida (*p* < 0.05). The same situation was noticed in the case of Gardnerella infection, which was also associated with a five times higher risk of having HPV than the group of women without Gardnerella (*p* < 0.05).

## 4. Discussion

According to international protocols and guidelines, HIV-positive women require special attention in terms of prevention, early detection, and diagnosis methods. Ellerbrock et al., (2000) suggested that as HIV-positive women live longer, many of them develop abnormal epithelial changes as cervical cancer precursors sometime during their evolution of HIV infection [2].

In our study, we found that 15% of HIV-positive women have cytological cervical precancerous lesions but a study conducted by Halichidis et al. in 2014 on nearly 100 HIV-positive women from Constanta Region (South-East Romania) identified that 54% of them had abnormal cytology [22]. The scores were much lower in women of a regional general population. Thus, Bosoteanu et al. (2011) found 7.59% abnormal cytology in 9269 women [23] and Stolnicu’s representative retrospective study from 2014 showed 5.9% abnormal cytology in more than 50,000 women screened from the national general population [24].

Massad et al., (2001) reported that 73% of HIV-positive women had at least one abnormal Pap test compared to 42% in HIV-negative women, during a 5-year follow-up [25]. Also, in our study, we found that HR-HPV types were detected in 47.5% of cases. Similar results were reported by Ene et al. in 2014, with 28 (43%) being HPV-infected out of 65 HIV-positive women [26]. Several regional studies also found relatively similar values of HR-HPV prevalence in general populations: Ursu et al. (2011) found 37.3% HPV-positive from 514 women in the general population [27], Feticu et al. (2012) found 54.4% HPV-positive women from 182 females from the Transylvania population [28], and Boda et al. (2016) reported 39.98% HR-HPV strains from 713 females of the general population [29].

The worldwide prevalence of HPV infection in women without cervical abnormalities is 11–12% with higher rates in sub-Saharan Africa (24%), Eastern Europe (21%), and Latin America (16%) [30]. We found 38.23% HPV-positive women with NILM.

Delmas et al. (2000), on behalf of the European Centre for the Epidemiological Monitoring of AIDS, reported that abnormal epithelial lesions were detected in the initial Pap test in 24.2% (113/467) of HIV-positive women [31].

Studies show that cases of persistent HPV infection in the absence of detectable cytological changes are frequent. We found that 68.42% of HIV-positive women have been detected with HPV infection in the absence of cytological cervical precancerous lesions, explicable by the persistence of HPV infection and leading to the necessity for meticulous monitoring. Ellerbrock’s findings indicate that the persistence of HPV infection in HIV-positive women conferred an increased risk for developing preinvasive cervical lesions and he found that an additional 26% (besides the initial 28%) will develop a cervical lesion after 54 months of follow-up [2].

Regarding HPV genotyping, we found that HPV 31 and 56 are the most frequent HPV types in HIV-infected women in the South East Region of Romania. The meta-analysis conducted by Clifford in 2017 [32] identified HPV 16 and 18 to be the most frequent HPV types in women living with HIV worldwide [32].

Women with CD4 count ≤ 200 cells/μL are significantly more likely to be infected with HPV despite long-term ART. Similar results were obtained by Ellerbrock, Palefsky, Johnson, and Vermund [2,33,34,35]. A detectable HIV load was found in 21% of HPV-positive women.

We detected a higher prevalence of HPV among sexually active women, even those with a single long-term partner, suggesting that sexual activity may be an important factor ininfection and the persistence of HPV infection. Also, the younger the age at which sexual life begins, the higher the risk of developing HPV infections. It seems that isolation of Candida or Gardnerella from vaginal discharge is more often noticed in HIV-positive women with HPV, than in HIV-positive women without HPV. However, it is well-known that HIV-infected women are more likely to have prevalent vaginal candidiasis, HR-HPV, and abnormal Pap cytology test [36].

Cervix infection with HPV and bacteria or fungi in correlation with the existence of HIV infection can lead to chronic inflammation, which favors HPV-related cervical precancerous lesions.

## 5. Conclusions

This descriptive study showed that the most frequent HPV type was 31 and the only cytological lesions were ASC-US and LSIL (all cases were HPV-positive). Women with CD4 < 200 cells/μL were more likely to be HPV-infected (nine times higher than the group with CD4 > 200 cells/μL). The risk of HPV infection was4.132 times higher for women with two or more sexual partners and 4.55 higher for those with an early sex life debut (under 18 years old). An association between Candida or Gardnerella infection with HPV was five times more frequent.

Other studies, on a larger group of HIV-positive women, have shown a higher percentage of association between HPV, vaginal infections, and cytology changes compared with our study. Studies conducted on general populations of women show a smaller percentage of those associations compared to this study.

Representing a small local study with a limited number of cases, further extensive studies are needed to achieve an appropriate conclusion.

The high prevalence of cervical precancerous lesions in HIV-positive women warrants the need for regular Pap cytology screening and co-testing for HPV detection in order to diagnose cervical cancer early when treatment could also be effective. This systematic screening for cytological premalignant cervical lesions must be continued for all women, especially for those with immunosuppression, combining HPV detection with Pap cytology as well as trying to include co-testing for HPV types in the National Screening Program.

## Figures and Tables

**Table 1:** Summary of factors correlated with detection of HR-HPV types.

Influencing Factors		Number of Women with HPV Detection		χ^2^_calc_	Odds Ratio	95% Confidence Interval		Significance (Two-Sided)
		Yes	No			Lower	Upper	
CD4	≤200 cells/μL	6	1	4.969	9.231	1.093	85.775	0.026
	>200 cells/μL	13	20					
HIV viral load	>51 copies/mL	4	1	2.420	5.333	0.539	52.734	0.120
	<50 copies/mL	15	20					
First sexual intercourse	≤18 years	14	8	5.105	4.550	1.181	17.524	0.024
	>18 years	5	13					
No. of sexual partners	>2 partners	15	10	4.177	4.125	1.102	16.667	0.041
	≤2 partners	4	11					
Vaginal candidiasis	Yes	9	3	5.199	5.4	1.183	24.645	0.023
	No	10	18					
Gardnerella	Yes	6	1	4.969	9.231	0.993	85.775	0.026
	No	13	20					

χ^2^_calc_: Chi-square coefficient performed for analized data.

**Table 2:** Association between HPV types with cytological results in HPV-positive women.

Case Number	HPV Types	PAP Cytology
04	56	LSIL
05	56	NILM
06	56	NILM
07	16, 31	LSIL
08	51	NILM
09	31	NILM
10	31, 56	ASCUS
11	18, 33	NILM
16	51, 52, 53, 56	NILM
17	31	NILM
18	31	NILM
24	31	NILM
25	56	NILM
27	68	ASCUS
33	39	LSIL
36	39, 53	ASCUS
35	31	NILM
37	53, 73	NILM
39	31, 52	NILM

## Data Availability

Not applicable.
